# Efficacy and Safety of Hyaluronic Acid Fillers for Horizontal Neck Lines: A Systematic Review and Meta-Analysis

**DOI:** 10.1093/asjof/ojaf163

**Published:** 2025-12-09

**Authors:** Zhen Yu Wong, Shradha Limbu, George Karanasios, Fabio Monks, Ryan Faderani, Muholan Kanapathy, Afshin Mosahebi

## Abstract

Horizontal neck lines are a common aesthetic concern among aging and younger populations. Hyaluronic acid (HA) fillers are increasingly used in this region, yet their effectiveness, safety, and optimal application strategies remain under-investigated. This systematic review and meta-analysis, conducted in accordance with PRISMA guidelines and registered with PROSPERO (CRD42024550863), aimed to evaluate the efficacy and safety of HA fillers for horizontal neck lines. Comprehensive searches of Ovid MEDLINE and Embase were performed up to May 2024. Eleven studies (*n* = 379) met the inclusion criteria. Pooled analysis demonstrated significant improvements in the Global Aesthetic Improvement Scale (mean, 1.75, 95% CI, 1.53-1.96) and wrinkle grading (mean difference, 1.43, 95% CI, 0.72-2.15). Instrumental assessments confirmed enhancements in hydration, elasticity, and dermal structure. The pooled retreatment rate within 12 months was 50%, and the complication rate 14%, with only mild, transient effects reported. Patient satisfaction was consistently high. In summary, HA fillers represent an effective and well-tolerated option for treating horizontal neck lines, producing sustained improvements in both aesthetic appearance and skin quality.

**Level of Evidence:** 4 (Therapeutic) 
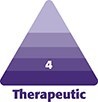

Aging of the neck is a multifactorial process characterized by dermal thinning, loss of elasticity, photodamage, and the progressive development of horizontal rhytides, commonly referred to as necklace lines.^1^ These lines are particularly prominent in elderly individuals and contribute to an aged appearance that is often incongruent with facial rejuvenation efforts. More recently, the demand for aesthetic treatment of the neck has grown, especially among younger individuals, as the phenomenon of “tech neck,” caused by frequent bending of the neck to look at digital devices, contributes to the earlier development of horizontal neck lines. The neck's delicate anatomy, including its thin dermis, limited subcutaneous fat, and high mobility, presents unique challenges for aesthetic interventions. Hyaluronic acid (HA) is a naturally occurring glycosaminoglycan present in various tissues such as the skin, connective tissue, and eyes. Its remarkable capacity to retain water contributes significantly to skin hydration and elasticity. As a major component of the extracellular matrix, HA also plays an essential role in providing structural support and maintaining the integrity of the skin.^2-4^ In dermatological practice, HA has garnered increasing interest for its aesthetic applications, particularly in softening facial wrinkles.^5^ Its benefits have been widely recognized in enhancing lip and cheek volume and addressing visible signs of skin aging.^6-8^

Although several individual studies have reported positive outcomes, the existing literature varies widely in terms of product type, treatment protocols, assessment methods, and follow-up durations in neck rejuvenation. Consequently, there remains a lack of robust, consolidated evidence on the overall effectiveness, durability, and safety of HA-based filler for horizontal neck lines in the aging population. This systematic review and meta-analysis seeks to critically evaluate the current evidence, synthesize subjective and objective outcome measures, assess adverse event profiles, and compare different HA formulations and delivery methods. The results aim to support clinicians in evidence-based decision making and highlight areas for further research in the aesthetic management of age-related neck changes.

## METHODS

### Information Source

This systematic review and meta-analysis was prospectively registered with the International Prospective Register of Systematic Reviews (PROSPERO) (CRD42024550863). The study was conducted in accordance with the Declaration of Helsinki. The study was exempt from IRB review because no confidential patient information was involved. The methodology followed the Preferred Reporting Items for Systematic Reviews and Meta-Analyses (PRISMA) 2020 guidelines (Appendix).^9^ A comprehensive search was conducted using Ovid MEDLINE from 1946 to May 23, 2024 and Embase from 1974 to May 23, 2024. The search strategy used a combination of keyword permutations related to HA in the neck. Furthermore, the reference lists of all included studies and relevant review articles were screened manually to identify further eligible studies.

### Study Selection and Eligibility Criteria

Studies were considered eligible for inclusion if they involved original research on human participants receiving HA-based injections as monotherapy specifically targeting horizontal neck lines. Eligible studies needed to report aesthetic or clinical outcomes, whether photographic, instrumental, or based on patient satisfaction, and had to be published in English in a peer-reviewed journal. Studies were excluded if they were case reports involving fewer than 5 patients, review articles, editorials, letters to the editor, conference abstracts, or if they lacked outcome data specific to horizontal neck lines. Two reviewers (Z.Y.W. and S.L.) independently screened all titles and abstracts for relevance, followed by full-text reviews of potentially eligible articles. Disagreements were resolved through discussion or, if necessary, by consultation with a third reviewer (G.K,). Final inclusion was based on consensus following full-text review.

### Data Extraction and Analysis

Data were extracted independently by 2 reviewers using a standardized data collection form. Extracted variables included study design, sample size, participant characteristics, type and formulation of HA filler used, injection technique and treatment protocol, number of sessions, follow-up duration, outcome measures, and reported adverse events. When clarification or additional information was required, the corresponding authors of the studies were contacted.

### Quality Assessment

Risk of bias was assessed independently by 2 reviewers using the Joanna Briggs Institute (JBI) critical appraisal tools for all included studies.^10^ Each study was evaluated across relevant methodological domains specific to its design (randomized or nonrandomized), and the overall risk of bias was categorized as low, moderate, or high. Any disagreements between reviewers were resolved through consensus.

### Data Extraction and Analysis

A qualitative synthesis of all included studies was first undertaken. Where quantitative data were sufficiently homogeneous, a meta-analysis was performed using the DerSimonian and Laird random-effects model to account for between-study variability.^11,12^ Continuous and proportional outcomes were pooled and reported as mean differences with 95% CIs. Heterogeneity was assessed using the *I*^2^ statistic, with values of 25%, 50%, and 75% considered to represent low, moderate, and high heterogeneity, respectively. All statistical analyses were conducted using STATA (Statacorp 17.0). Wrinkle grading and Global Aesthetic Improvement Scale (GAIS) scores were assessed as primary outcomes; improvements were reflected by a decrease of 1 to 2 points on wrinkle severity scales (lower scores indicating fewer wrinkles) and an increase of 1 to 2 points on the GAIS (higher scores indicating greater aesthetic improvement).

## RESULTS

### Study Selection

**Figure 1.**
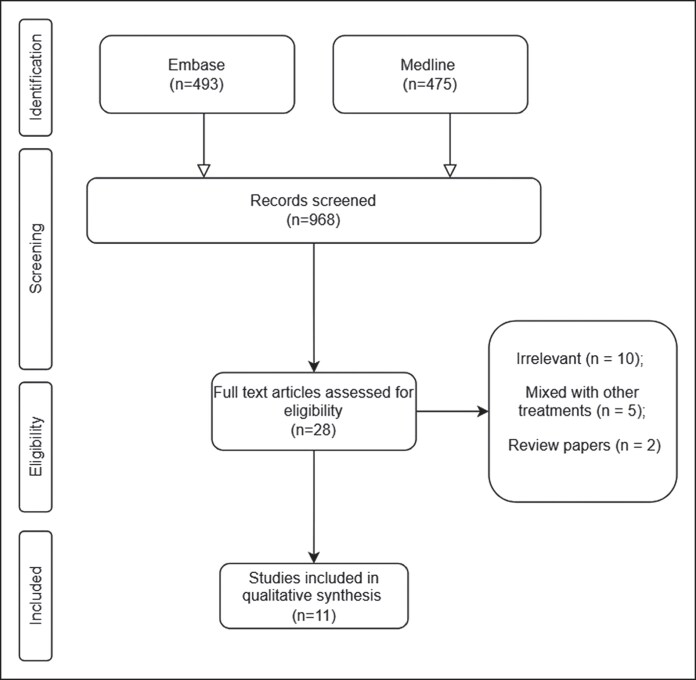
PRISMA flow diagram illustrating the process of study identification, screening, eligibility assessment, and inclusion for the systematic review and meta-analysis.

The initial database search identified 968 studies (475 from Ovid MEDLINE and 493 from Embase). After removing duplicates and screening titles and abstracts, 28 full-text articles were assessed. Eleven studies met the eligibility criteria and were included in the final analysis (Figure 1).^13-22^ Collectively, these studies enrolled 379 participants with horizontal neck lines treated with various formulations of HA.

### Study Characteristics

The included studies comprised 7 case series, 3 cohort studies, and 1 randomized controlled trial (Table 1). Study sample sizes ranged from 12 to 131, with a cumulative total of 379 patients. 360 out of 379 participants were female, with ages spanning from 20 to 75 years. Follow-up durations ranged from 3 weeks to 12 months. Crosslinked HA products included Belotero Balance (Merz Pharmaceuticals GmbH, Frankfurt, Germany), Restylane Vital and Restylane Refyne (Galderma, Uppsala, Sweden), Juvederm Volite VYC-12 (Allergan Aesthetics, an AbbVie company, Irvine, CA), and cohesive polydensified matrix HA (CPM-HA) fillers (Merz Pharmaceuticals GmbH). Hybrid HA complexes, such as Profhilo (IBSA Farmaceutici Italia Srl, Lodi, Italy), were employed in 3 studies, and noncrosslinked HA products including Redensity I (Teoxane Laboratories, Geneva, Switzerland) were used in 2 studies. Injection approaches were diverse but well-documented across studies: retrograde linear threading (*n* = 2 studies), minibolus or microdepot techniques (*n* = 2), 10-Point Bio Aesthetic Point Neck Technique using 29G needles (*n* = 2), linear threading or vertical injection with 31G 8 mm needles (*n* = 1), computerized jet injector with a 10 × 10 mm square-shaped tip at 70% pressure (*n* = 1), 25G 50 mm blunt cannula (*n* = 2), and 32G 4 mm ultra-thin wall needle (*n* = 1). Risk of bias was assessed using the JBI checklist and found to be low to moderate across most domains (Supplemental Tables 1-3).

**Table 1.** Summary of Included Studies

Study	Sample size	Country	Study design	Age	Gender (F:M)	Follow-up period	HA filler used	Type of HA filler	Injection method/device	Session protocol	Outcome measures and evaluation method
Bezpalko and Filipskiy^17^	15 (30)	Ukraine	Prospective cohort study	41	15:0	3 weeks	Redensity 1, Profhilo	Noncrosslinked HA, hybrid HA complex	25G 50 mm cannula	Two noncrosslinked HA products—Redensity 1 and Profhilo were injected into opposite sides of the face and neck at D0	Investigator-rated GAIS, ultrasound assessment of dermal thickness and echogenicity, and skin profilometry for roughness
Han et al^15^	12	Korea	Case series	54.7 (45-62)	8:4	2 months	HA solution	—	10 × 10 mm square-shaped tip at 70% pressure power	Up to four 0.15 mL injections at 4 week interval	Physician-assessed photographs and patient satisfaction survey at each session
Lee and Kim^19^	14 (28)	Korea	Retrospective Cohort study	45 (8)	14:0	2 months	Belotero Balance, Restylane Vital	Crosslinked HA	31G 8 mm needle with using either linear threading or vertical injection techniques	Three sessions at baseline, 1 month, and 3 months, each side treated with either Belotero Balance (monophasic) or Restylane Vital (biphasic). Up to 1 mL per side per session (average ≈0.8-0.9 mL)	Investigator- and patient-rated GAIS and satisfaction score; blinded photographic assessment
MacGillis and Vinshtok^18^	26	Canada	Retrospective cohort study	48.6	26:0	3 months	Esthélis Basic, nonanimal-derived noncrosslinked HA	Crosslinked HA, noncrosslinked HA	Computerized jet injector	Average 2.8 sessions per patient, spaced 4-6 weeks apart, using a computerized jet injector (EnerJet 2.0). Exact per-session volume not specified, but standard EnerJet ampoule = 1 mL HA	Corneometry for hydration, ultrasound imaging for dermal echogenicity, and surface profilometry for skin roughness
Niforos et al^23^	131	France	Case series	54 (32-72)	116:15	6 months	VYC-12/Juvederm Volite	Crosslinked HA	32G ultra-thin wall needles in multiple microdepot injections	D0, D30, and up to 1 touch-up injection at 9 months	Investigator-rated GAIS, patient satisfaction score, and skin elasticity (R2 parameter) through cutometry
Renga and Ryder^21^	50	Italy	Case series	55 (5.7)	50:0	12 months	VYC-12/Juvederm Volite	Crosslinked HA	32G needle and a minibolus technique	Two sessions: baseline (Day 0) and Month 9. 1 mL per session (total ≈2 mL)	Patient satisfaction (5-point Likert scale), investigator-assessed GAIS, and clinical photography
Rongthong et al. 2022^22^	30	Thailand	Case series	20-69	26: 4	6 months	Belotero Balance	Crosslinked HA	25G blunt cannula	3 mL injection at D0	Investigator-rated GAIS, patient satisfaction score, and photographic documentation at follow-up
Siperstein et al^16^	26 (52)	USA	Randomized trial	53.46 (35-75)	24:2	1 year	Restylane Refyne	Crosslinked HA	32G 4 mm needle with a retrograde linear threading technique vs 27G blunt cannula	Day 0: up to 2 cc total (average 0.86 mL) Day 30: up to 2 cc total (average 0.54 mL) for optimal treament Day 60: up to 4 cc (average 0.995 mL) for crossover from saline control group Total for trial: up to 4 mL (average 1.4 mL)	Investigator- and patient-rated GAIS, satisfaction score, and blinded panel review of standardized photographs
Sparavigna et al^13^	25	Italy	Case series	54 (41-65)	—	4 months	Profhilo	Hybrid HA complex	29G needle with 10-Point (Bio Aesthetic Point Neck Technique	2 mL injection at 4 week interval for 4 months	Investigator-rated GAIS, patient satisfaction score, and instrumental assessment using cutometry and corneometry
Sparavigna et al^14^	18	China	Case series	51 (38-60)	18:0	4 months	Profhilo	Hybrid HA complex	29G needle with 10-Point (Bio Aesthetic Point Neck Technique	2 mL injection at 4 week interval for 5 visits	Investigator-rated GAIS, patient satisfaction score, and objective measures including skin elasticity (cutometry) and hydration (corneometry)
Tseng and Yu^20^	32 (64)	Taiwan	ies	23-61	32:0	40 weeks	CPM-HA filler	Crosslinked HA	25G blunt cannulas	Day 0 injection with optional touch-up at 4 weeks; 1 mL per session (total up to 2 mL)	Investigator-rated GAIS and patient satisfaction, with photographic follow-up at 4 weeks and 3 months

CPM-HA, cohesive polydensified matrix hyaluronic acid; GAIS, Global Aesthetic Improvement Scale; HA, hyaluronic acid.

### Efficacy

**Figure 2.**
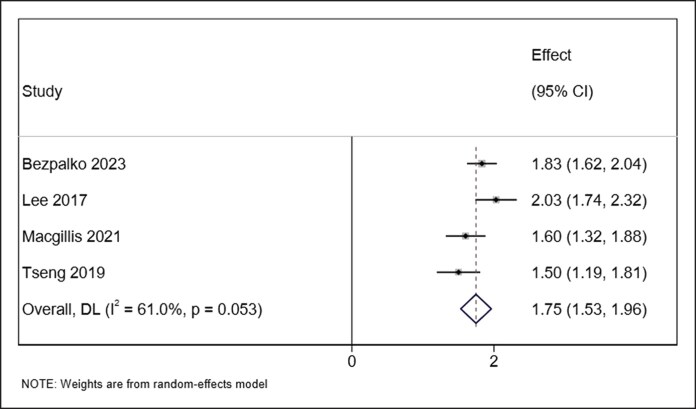
Forest plot showing pooled mean differences in Global Aesthetic Improvement Scale (GAIS) scores following hyaluronic acid (HA) filler treatment for horizontal neck lines. The *x*-axis represents mean GAIS scores with 95% CIs.

**Figure 3.**
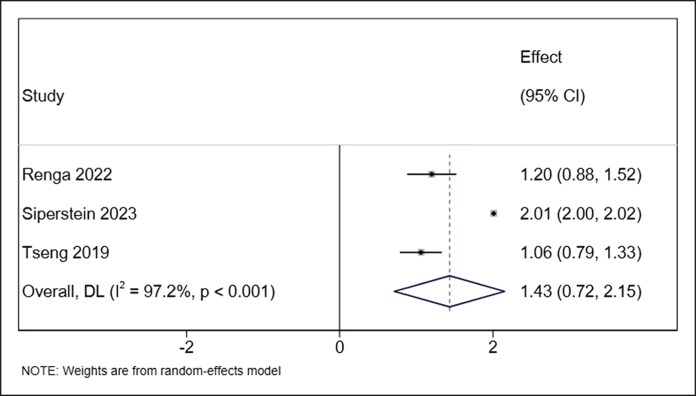
Forest plot showing pooled mean differences in patient satisfaction scores following HA filler treatment for horizontal neck lines. The *x*-axis represents satisfaction score differences with 95% CIs.

**Figure 4.**
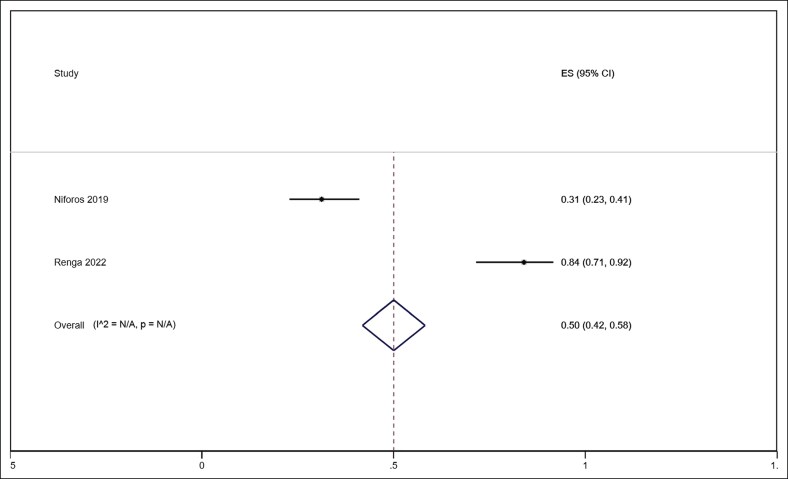
Forest plot showing pooled effect size of improvement in skin elasticity (R2 parameter) after HA filler treatment. The *x*-axis represents standardized mean difference with 95% CIs. ES, effect size.

Across the included studies, treatment with HA demonstrated consistent improvements in aesthetic and instrumental outcomes related to horizontal neck lines. Pooled analysis of 4 studies (number of patients: 110 patients) reporting the GAIS showed a significant subjective benefit, with a mean score of 1.75 (95% CI, 1.53-1.96, *I*^2^ = 61.0% *P* = .053) with no significant heterogeneity (Figure 2).^16,18-20^ Wrinkle severity, assessed in 3 studies (number of patients: 108 patients) using validated grading systems, demonstrated an improvement of 1.43 with significant heterogeneity (95% CI, 0.72-2.15, *I*^2^ = 97.2%, *P* < .001; Figure 3).^16,20,21^ One study (number of patients: 50 patients) reported a statistically significant improvement in patient-reported outcomes using the FACE-Q “Appraisal of the Neck” domain following HA treatment.^21^ These subjective results were supported by objective instrumental assessments. Skin hydration increased markedly at both superficial and deeper dermal levels as confirmed by corneometric evaluation, whereas skin roughness showed measurable reductions across all 3 profilometric parameters: Ra (average roughness), Rt (total height), and Rv (maximum depth).^18,23^ Ultrasound and histological analyses demonstrated increased dermal thickness and a localized reduction in echogenicity, indicative of enhanced hydration.^17^ Although echogenicity is an indirect indicator, when interpreted alongside corneometric data, it supports an overall improvement in skin quality. HA particles were transiently observed in the reticular dermis, dissipating by Week 3, yet signs of ongoing dermal stimulation and extracellular matrix remodeling persisted. Histological examination also revealed HA-induced disruption of collagen fibers, which may contribute to collagen remodeling and neocollagenesis. In terms of skin tone, instrumental colorimetric analysis showed increased brightness, reduced erythema, and mild pigmentation enhancement.^13^ Lastly, significant improvements in plastoelasticity were noted, with torsiometric measurements indicating greater skin turgor and improved immediate elastic recovery, particularly at later follow-up intervals.^13,14^ The need for repeat treatment was analyzed in a pooled meta-analysis of 2 studies (number of patients: 181 patients), showing that 50% (95% CI, 42%-58%, *I*^2^ = NA) of patients required retreatment within 12 months (Figure 4).^21,23^ Overall, patient satisfaction was also reported to be high across studies.

One randomized controlled trial involving 26 patients demonstrated that the HA filler Restylane Refyne significantly improved static horizontal neck rhytides when administered through either cannula or needle, with no serious adverse events reported.^16^ Notably, injections performed using needles were more effective than saline and showed superior outcomes compared with the cannula approach. Meanwhile, a cohort study with 26 patients reported that patients experienced slightly more intense pain with blunt cannulas than with sharp needles.^18^ Another study compared 2 injection techniques—linear threading technique (LTT) and vertical technique (VT)—as well as monophasic vs biphasic HA fillers.^19^ VT group achieved a mean GAIS score of 4.27 ± 0.75, compared with 3.60 ± 0.70 using LTT, demonstrating a statistically significant difference (*P* = .027). Correspondingly, patient satisfaction scores were higher in the VT group, supporting its superior aesthetic outcome at 2-month follow-up. Regarding formulation differences, a comparative study (25 patients) highlighted that high molecular weight (HMW) HA with lidocaine was associated with less injection site pain and better clinical outcomes at 3 weeks, whereas low molecular weight (LMW), high-concentration HA without lidocaine caused greater discomfort but initially produced a more pronounced lifting effect.^13^ However, the latter formulation degraded completely by 3 weeks and conferred no sustained benefit compared with the less concentrated HA.

### Complications

**Figure 5.**
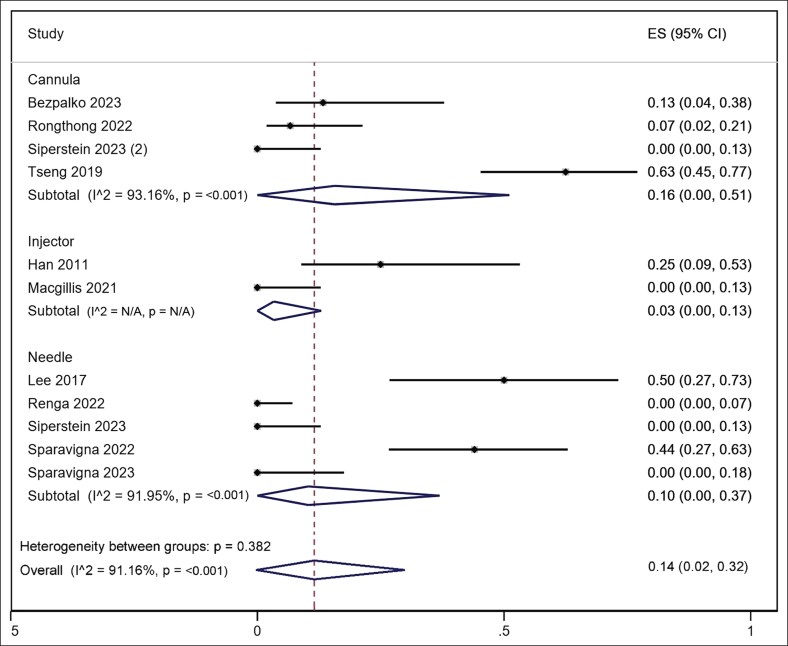
Forest plot of pooled complication rates stratified by delivery method (needle, cannula, or injector). The *x*-axis represents the proportion of participants experiencing complications shown with 95% CIs. Heterogeneity (*I*^2^) and subgroup *P*-values are reported. ES, effect size.

Ten studies reported safety data.^13-23^ The overall pooled complication rate was 14% (45/248, 95% CI, 2%-32%), with significant heterogeneity (*I*^2^ = 90.2%, *P* < .001; Figure 5). Most complications were mild and transient, including ecchymosis, edema, and local tenderness. Notably, 3 studies reported no adverse events. Six studies reported complications based on participant feedback, whereas 4 relied on investigator assessment.

Subgroup analysis by injection method demonstrated comparable safety profiles between groups (*P* = .382 for between-group heterogeneity). Needle-based techniques (*n* = 18/133) showed a pooled complication rate of 10% with significant heterogeneity (95% CI, 2%-37%, *I*^2^ = 92%, *P* < .001), whereas cannula-based approaches (*n* = 24/103) had a rate of 16% (95% CI, 0%-51% *I*^2^ = 93%, *P* < .001), and a lower rate in injector-based delivery (*n* = 3/38) yielded 3% (95% CI, 0%-13%, *I*^2^ = NA). Although complication rates appeared numerically lower with cannula and injector systems, the difference was not statistically significant.

## DISCUSSION

This systematic review and meta-analysis highlights HA filler as a clinically effective and well-tolerated approach for addressing horizontal neck lines. Beyond improving the appearance of static wrinkles, HA injections demonstrated multidimensional skin benefits including enhanced hydration, elasticity, and dermal structure. The consistency across patient-reported outcomes and objective measures such as dermal echogenicity and profilometric roughness suggests that HA exerts regenerative effects that go beyond temporary volume replacement. These changes are biologically meaningful, likely reflecting stimulation of fibroblast activity, extracellular matrix remodeling, and increased water retention within the dermis.

HA filler is increasingly regarded as a key modality in nonsurgical neck rejuvenation. In addition to high satisfaction rates, the treatment was associated with measurable structural improvements such as increased dermal thickness and collagen fiber modulation, as evidenced in histological evaluations.^24^ These findings are in line with prior work on facial aesthetics and support the evolving view of HA as a biostimulator rather than a purely volumizing agent. Notably, dermal improvement continued even after HA particles were no longer detected, underscoring the role of ongoing matrix activity. Improvements in skin brightness and reduction in erythema further suggest enhanced dermal health and vascular balance, contributing to the perception of youthfulness.

Although no serious adverse events were reported across the included studies, the neck presents unique anatomical risks that merit consideration. The region contains multiple superficial veins, perforating arterial branches, and a complex lymphatic network, making the risk of intravascular injection, vascular compromise, or lymphatic obstruction theoretically possible. Additionally, the thin dermis and mobility of the neck may predispose to filler migration or irregularities if injected too superficially or in large boluses. These potential risks underscore the importance of cautious technique, slow retrograde injection, and precise depth control when performing filler treatments in this area. Most studies in this review utilized intradermal or superficial subdermal injections, typically employing 29G to 32G needles or 25G cannulas. Needle-based delivery allows greater precision and controlled linear threading, whereas cannulas reduce puncture frequency and theoretically minimize bruising, albeit at the expense of accuracy in fine rhytid correction. Pneumatic injector systems, as explored by Han and MacGillis, provided more uniform product dispersion and reduced injection pain, although outcomes appeared modest compared with manual delivery.

When considering best practice for administration, this review highlights the importance of individualizing both technique and product choice.^25^ Although both needle and cannula techniques were effective, studies suggested sharper needles offered more precise results and reduced discomfort. Vertical injection approaches and the use of monophasic fillers were associated with higher GAIS and patient satisfaction scores. These outcomes may relate to improved diffusion and integration of the product. Additionally, HMW HA with lidocaine offered greater comfort and superior results compared with higher concentration LMW formulations that provided more initial lift but degraded faster.^26^ These findings reinforce the need for tailored planning based on anatomical features and product characteristics.

The longevity of effect averaged ∼14.4 months between initial and maintenance treatments, suggesting that HA filler is a durable option for patients seeking long-term outcomes. Although HA fillers have been extensively studied in the mid and upper face, these findings indicate that their effects are similarly long lasting when applied to the neck region.^27-29^ This parallel suggests that tissue biomechanics and filler rheology, rather than anatomical site alone, predominantly determine longevity and patient satisfaction. However, the anatomical complexity of the neck warrants consideration. With its thin dermis, high mobility, and proximity to vascular and lymphatic structures, the potential for filler migration must be taken seriously.^30-32^ Although no serious adverse events were reported, these theoretical concerns support the need for cautious techniques and thorough anatomical understanding during administration.^33^

Differences in HA crosslinking density and rheological properties may partly explain the variation in clinical outcomes observed between products. Highly crosslinked gels typically demonstrate greater longevity and lifting capacity but may be associated with higher injection resistance and potential for nodularity, whereas lightly crosslinked formulations are better suited for superficial placement and diffuse hydration. However, few studies directly compared crosslinking characteristics, and reporting of product rheology was inconsistent. However, the use of HA fillers for horizontal neck lines appears to be decreasing, coinciding with broader adoption of alternative rejuvenation approaches. Biostimulatory agents such as poly-L-lactic acid and calcium hydroxylapatite, as well as energy-based modalities including radiofrequency and microfocused ultrasound, are increasingly selected for their collagen-stimulating properties and longer-lasting effects. Despite this shift, HA fillers remain a safe, minimally invasive option for early-stage rhytid correction and can complement other treatments in combined rejuvenation protocols.

A key strength of this review lies in its comprehensive inclusion of both subjective and objective measures, offering a holistic understanding of HA efficacy. By integrating data from a randomized controlled trial and multiple prospective studies, the conclusions drawn are strengthened. Nonetheless, limitations exist. Variability in injection protocols and duration of follow-up limited the ability to formulate standardized guidance. For example, a follow-up of 3 weeks, as reported in 1 study, is insufficient to fully evaluate the persistence of treatment effect or late-onset adverse events, thereby limiting interpretation of long-term efficacy and safety. Moreover, the small number of comparative or randomized studies reduces the robustness of pooled estimates. A further limitation lies in the heterogeneity of outcome assessment. Most studies relied on subjective endpoints such as GAIS and patient satisfaction, which, while practical, are susceptible to recall and observer bias. Objective modalities—such as high-frequency ultrasound, cutometry, and colorimetry—were used in only a minority of studies, and there remains no validated, standardized scale for assessing neck rejuvenation outcomes. The development of core outcome sets and adoption of quantitative instruments would enhance comparability and methodological rigor in future research. Additionally, studies that reported patient intention to return for repeat treatment may not accurately reflect clinical need but rather patient preference. This highlights the importance of further research to determine the optimal timing for maintenance treatment and to assess whether repeat procedures are medically necessary or primarily driven by aesthetic expectations. Furthermore, this review was confined to the use of HA fillers for horizontal neck lines. Other filler classes such as calcium hydroxylapatite and poly-L-lactic acid, as well as alternative HA applications for skin quality improvement or contour refinement in the neck, were not included and may provide additional insight in future comparative analyses.

## CONCLUSIONS

HA filler is a safe and effective treatment option for horizontal neck lines, demonstrating significant improvements in wrinkle severity, skin hydration, elasticity, and patient satisfaction. The pooled complication rate was low and limited to mild, transient effects, confirming a favorable safety profile. Although current evidence supports its clinical utility, variability in study design and follow-up duration underscores the need for further high-quality research to define optimal injection protocols and long-term outcomes.

### Supplemental Material

This article contains supplemental material located online at https://doi.org/10.1093/asjof/ojaf163.

## Supplementary Material

ojaf163_Supplementary_Data
