# Supplementary material for: Efficacy and Safety of Hyaluronic Acid Fillers for Horizontal Neck Lines: A Systematic Review and Meta-Analysis
Source: Aesthet Surg J Open Forum. 2025 Dec 9;8:ojaf163. doi: 10.1093/asjof/ojaf163 (PMC12813632; doi:10.1093/asjof/ojaf163)
Supplement: ojaf163_Supplementary_Data [file ojaf163_supplementary_data.zip › Supplemental Table 1.docx]

**Supplemental Table 1**. Joanna Briggs Institute (JBI) Critical Appraisal Checklist for Case Series

| Study | Q1 | Q2 | Q3 | Q4 | Q5 | Q6 | Q7 | Q8 | Q9 | Q10 | Overall |
| --- | --- | --- | --- | --- | --- | --- | --- | --- | --- | --- | --- |
| Rongthong 2022 | 1 | 1 | 1 | 1 | 1 | 1 | 1 | 1 | 1 | 1 | Low |
| Renga 2022 | 1 | 1 | 1 | 1 | 1 | 0 | 1 | 1 | 1 | 1 | Low |
| Niforos 2019 | 1 | 1 | 1 | 1 | 1 | 0 | 0 | 1 | 1 | 1 | Low |
| Tseng 2019 | 1 | 1 | 1 | 1 | 1 | 0 | 0 | 1 | 1 | 1 | Low |
| Han 2011 | 1 | 1 | 1 | 1 | 1 | 0 | 0 | 1 | 1 | 1 | Low |
| Sparavigna 2023 | 1 | 1 | 1 | 1 | 1 | 0 | 0 | 1 | 1 | 1 | Low |
| Sparavigna 2022 | 1 | 1 | 1 | 1 | 1 | 0 | 0 | 1 | 1 | 1 | Low |
| Q1) Were there clear criteria for inclusion in the case series? Q2) Was the condition measured in a standard, reliable way for all participants included in the case series? Q3) Were valid methods used for identification of the condition for all participants included in the case series? Q4) Did the case series have consecutive inclusion of participants? Q5) Did the case series have complete inclusion of participants? Q6) Was there clear reporting of the demographics of the participants in the study? Q7) Was there clear reporting of clinical information of the participants in the study? Q8) Were the outcomes or follow up results of cases clearly reported? Q9) Was there clear reporting of the presenting site(s)/clinic(s) demographic information Q10) Was statistical analysis appropriate?"  1 = criterion met; 0 = criterion not met | | | | | | | | | | | |
