# Supplementary material for: Efficacy and Safety of Hyaluronic Acid Fillers for Horizontal Neck Lines: A Systematic Review and Meta-Analysis
Source: Aesthet Surg J Open Forum. 2025 Dec 9;8:ojaf163. doi: 10.1093/asjof/ojaf163 (PMC12813632; doi:10.1093/asjof/ojaf163)
Supplement: ojaf163_Supplementary_Data [file ojaf163_supplementary_data.zip › Supplemental Table 2.docx]

**Supplemental Table 2**. Joanna Briggs Institute (JBI) Critical Appraisal Checklist for Randomised Controlled Trials

| Study | Q1 | Q2 | Q3 | Q4 | Q5 | Q6 | Q7 | Q8 | Q9 | Q10 | Q11 | Q12 | Q13 | Overall |
| --- | --- | --- | --- | --- | --- | --- | --- | --- | --- | --- | --- | --- | --- | --- |
| Siperstein 2023 | 1 | 1 | 1 | 1 | 1 | 1 | 1 | 1 | 1 | 1 | 1 | 1 | 1 | Low |
| Q1) Was true randomisation used for assignment of participants to treatment groups? Q2) Was allocation to treatment groups concealed? Q3) Were treatment groups similar at the baseline? Q4) Were participants blind to treatment assignment? Q5) Were those delivering treatment blind to treatment assignment? Q6) Were outcomes assessors blind to treatment assignment? Q7) Were treatment groups treated identically other than the intervention of interest? Q8) Was follow-up complete and if not, were differences between groups in terms of their follow-up adequately described and analysed? Q9) Were participants analysed in the groups to which they were randomised? Q10) Were outcomes measured in the same way for treatment groups? Q11) Were outcomes measured in a reliable way? Q12) Was appropriate statistical analysis used? Q13) Was the trial design appropriate, and any deviations from the standard RCT design (individual randomisation, parallel groups) accounted for in the conduct and analysis of the trial?  1 = criterion met; 0 = criterion not met | | | | | | | | | | | | | | |
