# Supplementary material for: Efficacy and Safety of Hyaluronic Acid Fillers for Horizontal Neck Lines: A Systematic Review and Meta-Analysis
Source: Aesthet Surg J Open Forum. 2025 Dec 9;8:ojaf163. doi: 10.1093/asjof/ojaf163 (PMC12813632; doi:10.1093/asjof/ojaf163)
Supplement: ojaf163_Supplementary_Data [file ojaf163_supplementary_data.zip › Supplemental Table 3.docx]

**Supplemental Table 3**. Joanna Briggs Institute (JBI) Critical Appraisal Checklist for Cohort Studies

| Study | Q1 | Q2 | Q3 | Q4 | Q5 | Q6 | Q7 | Q8 | Q9 | Q10 | Q11 | Overall |
| --- | --- | --- | --- | --- | --- | --- | --- | --- | --- | --- | --- | --- |
| Lee 2017 | 1 | 1 | 0 | 0 | 1 | 0 | 0 | 1 | 1 | 1 | 1 | Moderate |
| Macgillis 2021 | 1 | 1 | 0 | 0 | 1 | 1 | 1 | 1 | 1 | 1 | 1 | Low |
| Bezpalko 2023 | 1 | 1 | 0 | 0 | 1 | 1 | 1 | 1 | 1 | 1 | 1 | Low |
| Q1) Were the two groups similar and recruited from the same population? Q2) Were the exposures measured similarly to assign people to both exposed and unexposed groups? Q3) Was the exposure measured in a valid and reliable way? Q4) Were confounding factors identified? Q5) Were strategies to deal with confounding factors stated? Q6) Were the groups/participants free of the outcome at the start of the study (or at the moment of exposure)? Q7) Were the outcomes measured in a valid and reliable way? Q8) Was the follow-up time reported and sufficient to be long enough for outcomes to occur? Q9) Was follow-up complete, and if not, were the reasons for loss to follow-up described and explored? Q10) Were strategies to address incomplete follow-up utilised? Q11) Was appropriate statistical analysis used?  1 = criterion met; 0 = criterion not met | | | | | | | | | | | | |
